# Cognitive load in switching between egocentric and allocentric spatial frames of reference: a pupillometry study

**DOI:** 10.1038/s41598-025-11122-7

**Published:** 2025-07-21

**Authors:** Renato Orti, Tina Iachini, Eleonora D’Agostino, Francesco Ruotolo, Gennaro Ruggiero

**Affiliations:** Laboratory of Cognitive Science and Immersive Virtual Reality, CS-IVR, Department of Psychology, University of Campania L. Vanvitelli, Viale Ellittico, 31, 81100 Caserta, Italy

**Keywords:** Egocentric allocentric frames of reference, Visuo-spatial switching processes, Cognitive load, Pupillometry, Psychology, Cognitive neuroscience, Attention, Spatial memory

## Abstract

Every day we combine and switch between body-centered (egocentric) and object-centered (allocentric) spatial representations. Several studies so far have reported a greater difficulty to switch from an allocentric reference frame to an egocentric one than vice-versa. To clarify this effect, the present work measured the cognitive load underlying switching vs. non-switching processes between reference frames through cognitive pupillometry. Participants performed a custom-designed visuo-spatial memory task, while pupil dilation variations were measured with eye-tracking. After memorizing triads of objects, participants provided judgments of relative distance in non-switching (only-egocentric, only-allocentric) and switching (from-ego-to-allo, from-allo-to-ego) conditions. The results showed a larger pupil dilation in switching judgments from-allocentric-to-egocentric reference frames than from-egocentric-to-allocentric ones. Moreover, pupil evoked-responses were also larger in allocentric- than egocentric-based non-switching conditions. Overall, the results showed that, for both non-switching and switching visuo-spatial processes, starting from an allocentric-based representation elicits a higher cognitive load than starting from an egocentric-based one. Thus, the disproportional effort in visuo-spatial switching processes seems to be determined by the first reference frame adopted that, in turn, contaminates also the following one.

##  Introduction

During a meal one could encode the position of the fork with respect to oneself (i.e., egocentrically), but also with respect to the plate where the food is (i.e., allocentrically). This example illustrates how we encode the position of objects in the environment either using a body-centered (egocentric) or an object-centered (allocentric) spatial representations ^1–4^. The example further illustrates that we constantly switch between body- and object-centred spatial representations to achieve various everyday goals. In other words, rather than relying on a single reference frame, we translate spatial representations between them ^5–7^.

A growing body of neuroimaging studies pointed to a fronto-parietal network subserving egocentric representations, and to medio-temporal and parieto-temporal regions subserving allocentric ones ^8–13^. The visuospatial translational processes between spatial representations are supposed to be supported by postero-medial brain regions like retrosplenial and posterior cingulate cortices (RSC, PCC)^14,15^, by the Locus-Coeruleus Noradrenaline system (LC-NA) with the prefrontal cortex (PFC) ^16,17^, and also by frontal brain regions (Superior and Middle Frontal Gyri) beside the Temporo-Parietal Junction ^18^.

Behaviourally, several studies have suggested an imbalance in the effort needed to switch between egocentric- and allocentric-based spatial strategies in healthy populations of young and elderly adults ^18–22^, as well as in clinical populations (for pathological aging see ^6,23^; for psychiatric disorders see^24^). In this regard, Harris and colleagues^19^ compared healthy young and elderly participants on a visuospatial navigation task requiring them to alternatively adopt a response (egocentric) or place (allocentric) spatial strategy. The authors found that, when participants were required to switch towards an allocentric-based spatial strategy, elderly participants were less accurate and took longer compared to young adults. Conversely, such difference did not emerge when participants were required to adopt an egocentric-based spatial strategy (for similar results see also^25^). Overall, the authors linked such age-related visuo-spatial switching difficulty to physiological changes affecting alternatively, but not exclusively, brain systems devoted to switching processes per se (i.e., LC-NA PFC system), or underlying allocentric-based visto-spatial processes (e.g., medio-temporal brain structures)^19,20^.

More recently, Orti and colleagues ^22^ found an imbalance in the effort needed to switch between spatial representations also in healthy young adults (see also^18,21^). The authors administered an ad hoc devised visuo-spatial memory task (the ‘Ego-Allo Switching task’ ^6,7^), requiring participants to memorize triads of geometrical objects, then to provide switching (from-ego-to-allo, from-allo-to-ego) and non-switching (only-egocentric, only-allocentric) spatial judgments of relative distances about the memorized stimuli. The results showed that participants were overall slower and less accurate in switching from an allocentric to an egocentric reference frame compared to switching in the opposite direction, namely from an egocentric to an allocentric reference frame. In both cases, however, egocentric judgments were more accurate and faster than allocentric ones. This indicates a facilitation for the egocentric component over the allocentric one, especially when the body constitutes the first egocentric frame.

Interestingly, a similar pattern of results was also found in clinical populations with a diagnosis of dementia ^6,23^. Specifically, Ruggiero and colleagues ^6^ compared visuo-spatial switching performances of patients with a diagnosis of amnestic Mild Cognitive Impairment (aMCI) and early Alzheimer disease (eAD) with normal controls (NCs) by means of the ‘Ego-Allo Switching Task’. They found that the aMCI and eAD groups were less accurate compared to NCs in switching from an allocentric towards an egocentric spatial frame. Interestingly, the performance of the aMCI group did not differ from those of NCs when they were required to switch, instead, from an egocentric to an allocentric reference frame; differently, the eAD group exhibited a significant poorer performance also in switching from an egocentric to an allocentric reference frame compared to both NCs and aMCI groups. The authors speculated that the neat impairment shown by aMCI and eAD patients in visuo-spatial switching processes from allocentric to egocentric reference frames could represent a particularly resource demanding process for a deteriorating brain, that could also differentiate between atypical and typical visuo-spatial processes ^6^. Moreover, visuo-spatial switching processes between both reference frames seems to be wholly impaired only in the advanced stages of pathological aging (i.e., eAD; ^6^).

Altogether, a common element seems to emerge in previous studies: the higher difficulty in visuo-spatial switching processes appears to be associated with the allocentric component ^19,22,25^. In this view, the allocentric component may constitute the primary source of difficulty in visuo-spatial switching processes. Switching between different tasks or strategies is a resource demanding process that produces a ‘switch cost’ that witnesses to the involvement of a different cognitive load ^26–29^. Such switch cost could be due to endogenous or exogenous reorganizational processes needed to disengage from the actual task or strategy in favour of a different one ^26^.

Preliminary evidence suggesting unequal cognitive load in visuo-spatial switching processes was provided from a recent fNIRS study aimed to investigate the cortical correlates of visuo-spatial switching and non-switching processes ^18^. Specifically, it has been shown that switching processes from an allocentric to an egocentric representation rely more on frontal brain regions (superior, middle and inferior frontal gyri) compared to switching processes from an egocentric to an allocentric representation ^18^. A similar larger involvement of frontal regions (superior and middle frontal gyri) was also found in allocentric- compared to egocentric-based non-switching spatial representations. On the whole, the spread involvement of frontal regions in allocentric-based switching and non-switching processes could be symptomatic of a more resource demanding process *per se* ^6,7^;^22^.

While the literature reviewed so far provides a behavioural and neurofunctional body of proofs suggesting an imbalance in the effort needed to switch between egocentric- and allocentric-based spatial frames, to the best of our knowledge a psychophysiological measure of the cognitive load underlying visuo-spatial switching processes is still lacking. Such a measure should complement response times and accuracy data to increase the robustness of previous findings. In this regard, a useful tool for measuring the cognitive load is the ‘cognitive pupillometry’, i.e. the measurement of the pupil size (or pupil dilation variations) during cognitive processes ^30–32^. Typically, pupil size diameter increases proportionally to the increase of task demands (for a review see ^33–35^). This technique has demonstrated its efficacy also in measuring the cognitive effort behind the adoption of allocentric-based navigational strategies (i.e., survey) ^36^. Therefore, cognitive pupillometry can enrich the interpretation of behavioural data and offer additional insights into the cognitive processes involved in egocentric–allocentric transformations. Furthermore, task-evoked changes in pupil size have been closely linked to the activity of the locus coeruleus–noradrenaline (LC-NA) system, a neuromodulatory network implicated in attention regulation, effort allocation, and flexible cognitive control, including the ability to switch between competing strategies^16,37^.

Taken together, the studies presented above raise the question of whether the switch cost could be due to a high cognitive load related to the translational processes per se (namely to the disengagement from a spatial strategy in favour of another one), or to an imbalance in the cognitive resources needed to switch from a specific reference frame (i.e., allocentric).

Here, we aimed to measure the cognitive load underlying switching and non-switching processes between egocentric and allocentric reference frames through pupil dilation variations, beside behavioural measures like mean of accuracy and response times. Participants underwent to the ‘Ego-Allo Switching task’ ^6,7,18,22^. They were required to memorize triads of geometrical objects, then to provide two spatial judgments of relative distance in switching and non-switching conditions. In the non-switching conditions, the two spatial judgments regarded the same reference frames, i.e. only-egocentric (Ego-Ego) or only-allocentric (Allo-Allo). In the switching conditions, instead, the two spatial judgments required a switch from-egocentric-to-allocentric (Ego-Allo) or alternatively from allocentric-to-egocentric (Allo-Ego) reference frames. During the task, pupil dilation variations were measured by means of a wearable eye-tracking system. This experimental paradigm was based on previous studies dealing with healthy adults ^38–40^, neurological patients ^6,41^, as well as blind people ^7^, and has proved its efficacy in distinguishing between spatial reference frames.

Regarding pupil evoked responses, we expected larger pupil dilation variations in the switching than in the non-switching conditions, and in allocentric (Allo-Ego) than egocentric (Ego-Allo) switching conditions. Moreover, a greater pupil dilation is also expected in allocentric (Allo-Allo) than egocentric (Ego-Ego) non-switching spatial judgments. With regard to behavioural performance, in line with previous literature, we expected lower accuracy and higher response times in switching than non-switching conditions ^6,19^. Allo-Ego switching processes should be slower and less accurate than Ego-Allo ones ^6,18,22^. In a similar way, allocentric-based non-switching processes (Allo-Allo) should be slower and less accurate compared to egocentric-based ones (Ego-Ego) ^18,42^.

##  Materials and methods

###  Participants

The appropriate sample size for the experiment was determined through an a-priori power analysis with G*Power, version 3.1.9.4 ^43^ with the following parameters: Cohen’s effect size f = 0.65, α = 0.05, Power (1-b) = 0.95, N. of groups = 1, N. of measurements = 4 (Spatial Judgments: Ego-Ego, Allo-Allo, Ego-Allo, Allo-Ego), correlation among measurements = 0.5. The minimum total sample size was 16.

Twenty participants (10 females) aged between 18 and 28 years (M_age_ = 23, SD_age_ = 3.21; M_education_ = 16.35, SD_education_ = 2.62) were recruited for the study. All participants had normal or corrected-to-normal vision, no reported motor, sensory, neurological or psychiatric disorders, and were all right-handed as assessed by the Edinburgh Handedness Inventory ^44^ (EHI score > 0.5). Each participant gave informed consent to take part in the study. Participants were recruited and tested in accordance with the requirements of the relevant local ethics committee and the 2013 Declaration of Helsinki. The study then received official approval from the ethics committee of the Department of Psychology at the University of Campania “Luigi Vanvitelli” (ethics approval number 7/2024).

### Stimuli

**Fig. 1:**
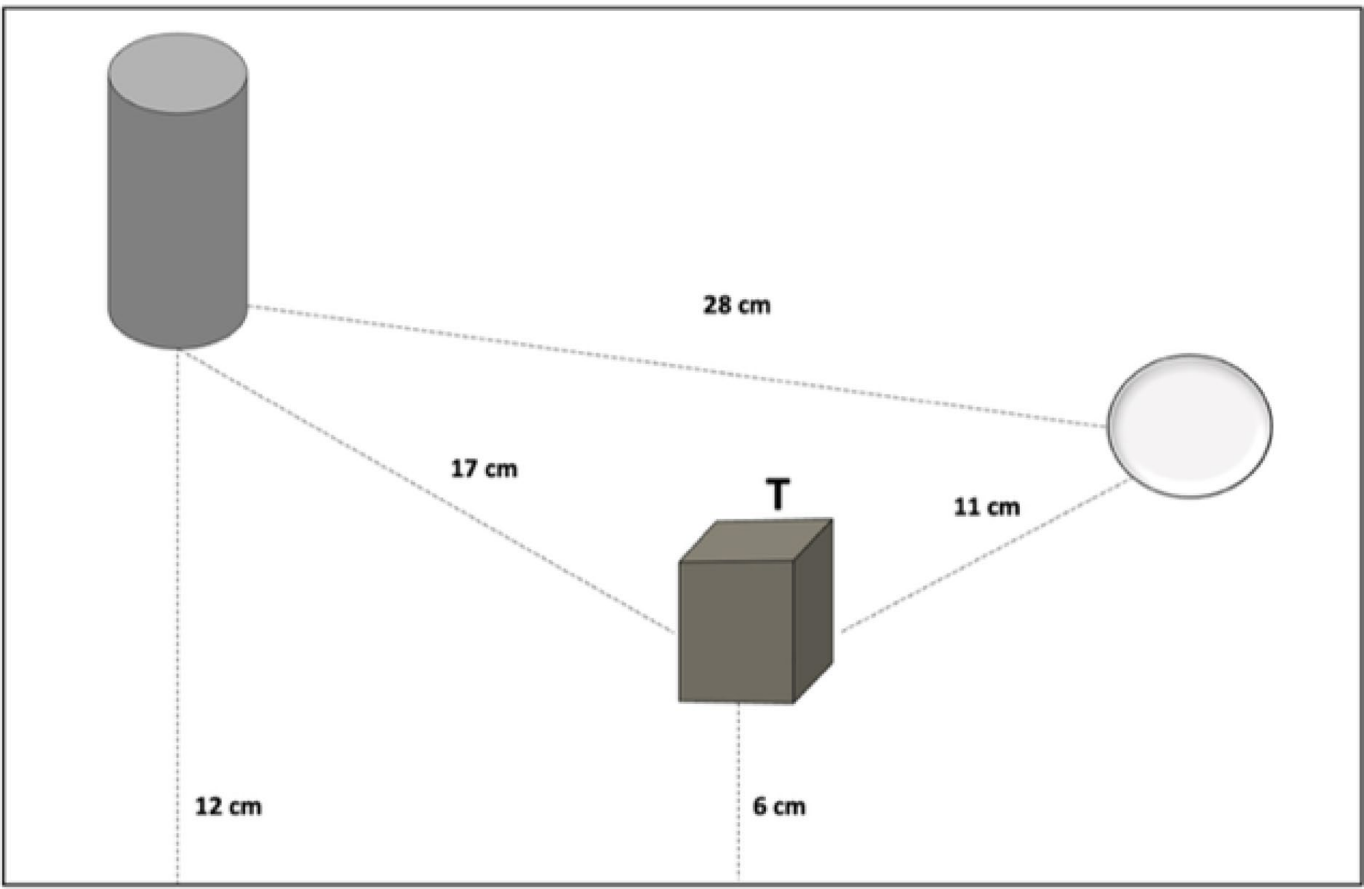
Task layout. Schematic example of the task layout where “T” represents a target-object (e.g., the cube) that is the point of reference used to provide the allocentric judgments. Black dashed lines indicate relative distances between object-to-object (e.g., sphere-cube) and subject-to-object distances.

A virtual version of the ‘Ego-Allo Switching Task’ ^6,7,38^ was developed by using SketchUp Make (Trimble, USA). Six 3D geometric objects (cone, cylinder, cube, parallelepiped, pyramid, sphere) of large (8 × 8 cm, but parallelepiped and cylinder 8 × 11 cm) and small size (6 × 6 cm, but parallelepiped and cylinder 6 × 9 cm) were designed and arranged in two series (A & B) and presented on twenty-four textured plasterboard panels (each measuring 50 × 30 × 2 cm). The panels were presented centrally in front of the participants. Each triad was arranged according to the following criteria: (i) the distances between the objects were clearly perceived; (ii) the level of metric difficulty in comparing egocentric and allocentric distances was the same for all judgments; (iii) each triad was presented aligned with the participants’ midsagittal plane. For an example of the task layout see Fig. 1. In this case, the distances between the stimuli were: cube-sphere = 11 cm, sphere-cylinder = 28 cm, cylinder-cube = 17 cm. The cube and the cylinder were respectively 6 cm and 12 cm from the edge. The cube was the target, i.e., the reference point for the allocentric judgments. The metric difference between the two objects closest to the body (12 − 6) and the two objects closest to the sphere (17 − 11) was the same, i.e., 6 cm. The luminance of the stimuli was kept constant (mean luminance across stimuli = 10.5 lux).

### Setting

**Fig. 2:**
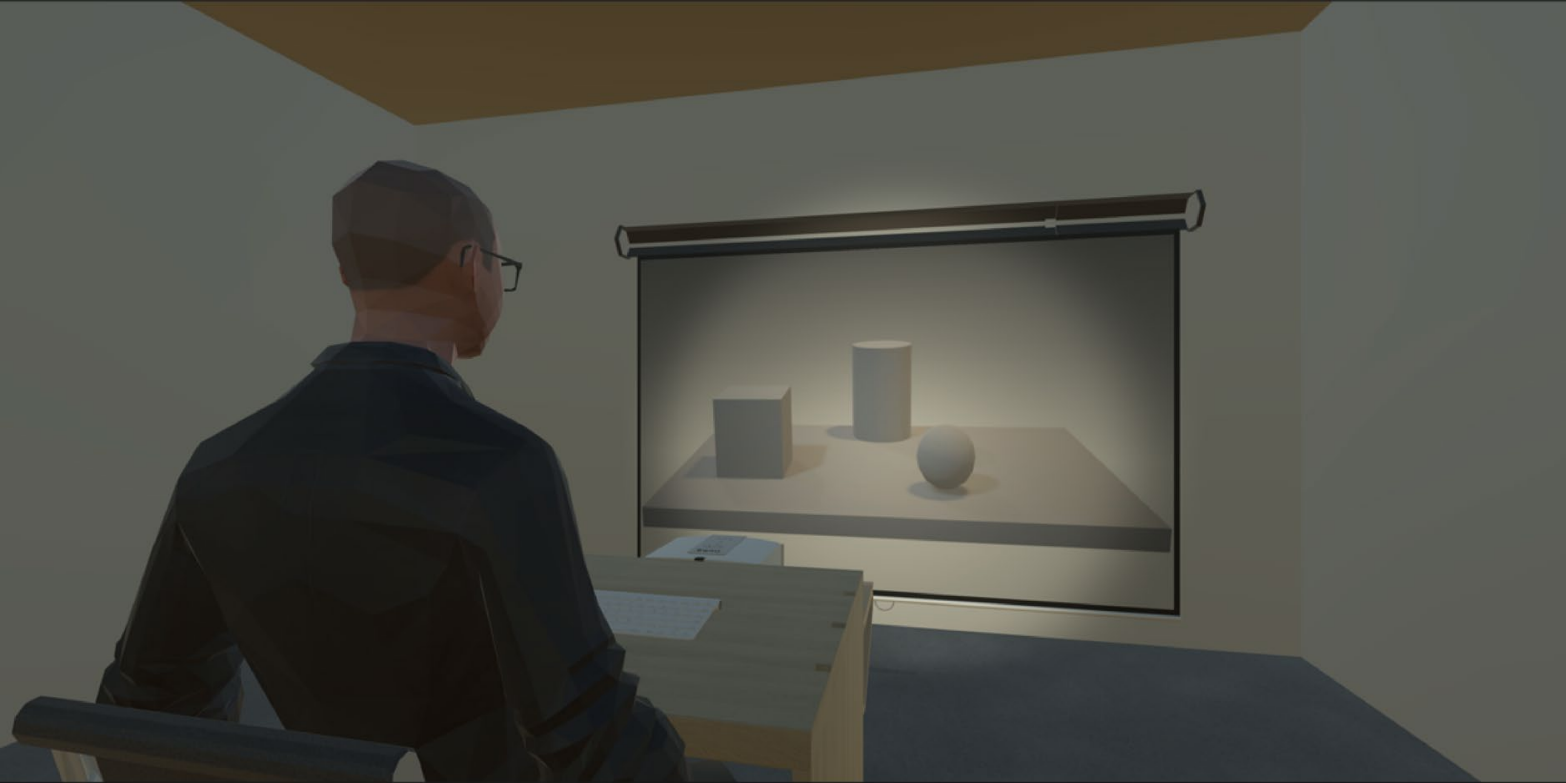
Example of the experimental setup: the participant sat behind a desk on which a keyboard (in white in the figure) was placed for the collection of motor responses. A video projector was positioned in front of the desk, projecting stimuli on the wall in front of the participant (~ 360 cm). The luminance of the environment was kept constant and matched to that of the stimuli (M = 9.3 lux). The participant wore the Tobii Pro Glasses 2 eye tracking system (i.e. the participant’s glasses in the picture) to continuously measure left and right pupil size changes. The picture was created through the Unity graphic engine software (Unity Technologies), version 2022.3.21f1 (https://unity.com).

The experiment took place in a soundproof room of the Cognitive Science and Immersive Virtual Reality Laboratory (CS-IVR, Department of Psychology, University of Campania “L. Vanvitelli”, Italy; see Fig. 2 for a reproduction of the experimental setting). The stimuli were presented centrally in front of the participants on the wall *via* a video projector (Fig. 2). The projection had a resolution of 1024 × 768 pixels, a refresh rate of 60 Hz and measured 116 × 76 cm. Participants sat in a chair in front of a desk at a distance from the projection of around 360 cm. The visual angle of the projection spanned horizontally for about 1.8° and vertically for around 1.57° resulting in a foveated stimulus projection (https://www.sr-research.com/visual-angle-calculator). The experiment was set up and run using the PsychoPy software (v 2022.2.5)^45^. The luminance of the setting was kept constant and matched with those of the stimuli (mean luminance = 9.3 lux).

### Procedure

#### Training phase

Before starting the experiment, participants were given written instructions about the task, which were orally revised: they were asked to memorize the objects and their relative positions as accurately as possible. At the beginning of the experimental session, the 3D geometric objects used in the experiment were presented one by one, and participants were asked to name them aloud to avoid possible naming problems. During a training phase lasting for 5 min, participants familiarized with the entire experimental procedure. The learning phase began as soon as participants reported that they had fully understood the task.

#### Learning phase

**Fig. 3:**
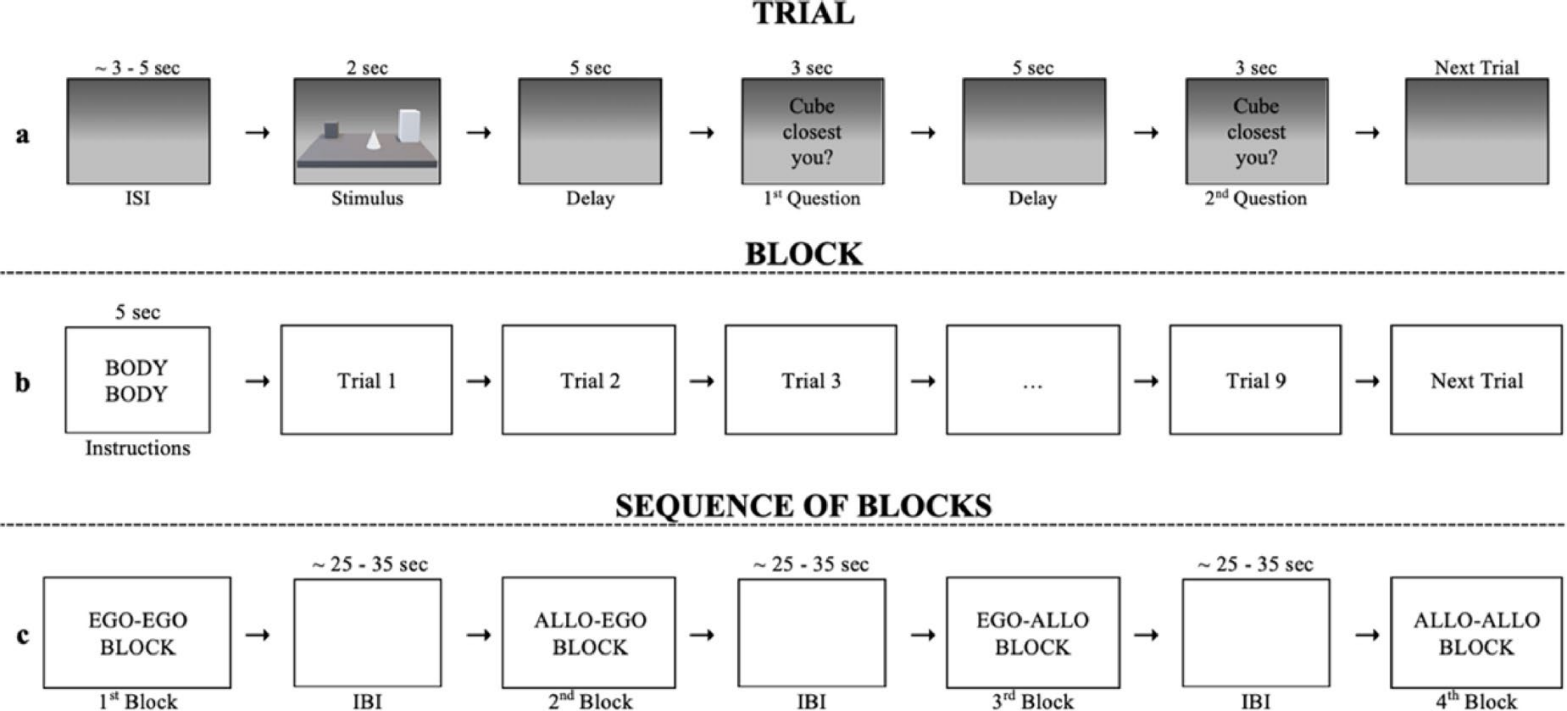
Experimental flow. The figure shows an example of trial (**a**), block (**b**) and sequence of blocks (**c**). **a** Each trial started with an ISI (random duration between 3–5 s). The stimulus was presented for 2 s and followed by a delay of 5 s (i.e., learning phase). Two spatial judgments were required subsequently. Each question appeared for 3 s, during which participants had to provide spatial judgment through motor response. A second delay of 5 s was presented between 1st and 2nd spatial judgments (i.e., testing phase). **b** Each block started with a brief instruction presented for 5 s informing the participant about the pair of spatial judgments to provide. The following instructions could appear: BODY–BODY (two egocentric spatial judgments); OBJECT–OBJECT (two allocentric spatial judgments); BODY–OBJECT (an egocentric then an allocentric spatial judgment); OBJECT–BODY (an allocentric then an egocentric spatial judgment). **c** Each sequence included the four spatial tasks (i.e., Ego-Ego, Allo-Allo, Ego-Allo, Allo-Ego) that were presented in a pseudo-randomized order across participants.

Participants were asked to memorize the objects and their positions presented for 2 s. Then the objects disappeared, and after a 5 s delay during which a blank screen was shown, the testing phase began (see Fig. 3a for a schematic representation of the experimental procedure).

#### Testing phase

Participants were asked to provide egocentric and allocentric spatial judgments of relative distances via motor responses (pressing on keyboard buttons that were counterbalanced across participants). The egocentric questions were of the type: “*Was object X the closest to you?*”; the allocentric questions were of the type “*Was object X the closest to object Y*?”. Both egocentric and allocentric questions were presented in a shorter form (e.g., egocentric: “*X closest you?*”; allocentric: “*X closest Y*?”). The questions appeared in front of the participants and lasted for 3 s during which participants gave a motor response by pressing the key assigned to “Yes” or “No” (the keys assigned to “Yes” and “No” were counterbalanced across participants).

To assess switching and non-switching judgments for each triad, participants had to make two spatial judgments in succession. For the switching condition, two judgments were required that involved two different anchor points (e.g., from-egocentric-to-allocentric and from-allocentric-to-egocentric), whereas for the non-switching condition, two judgments were required that involved the same frame of reference (e.g., from-egocentric-to-egocentric or from-allocentric-to-allocentric). Thirty-six triads were presented, 18 for the switching conditions and 18 for the non-switching conditions. The 18 switching triads were associated with 36 questions, 18 of which required a switch from an egocentric to an allocentric reference frame (Ego-Allo block) and 18 of which required a switch from an allocentric to an egocentric reference frame (Allo-Ego block). The 18 non-switching triads were associated with 36 questions, 18 of which required two subsequent egocentric (Ego-Ego) spatial judgments and 18 of which required two subsequent allocentric (Allo-Allo) spatial judgments. Triads were associated with questions in counterbalanced order and presented randomly. This controlled for material and order effects.

A block design was used for stimulus presentation and four blocks of nine trials each were presented: two switching blocks (Ego-Allo, Allo-Ego), two non-switching blocks (Ego-Ego, Allo-Allo) (see Fig. 3b). In order to homogenise the level of difficulty of the four blocks, each block was preceded by a short 5 s instruction aimed at informing the participant about the pair of spatial judgments he/she had to make (e.g. “BODY-BODY” for two egocentric spatial judgments, i.e. the “Ego-Ego block”; “OBJECT-OBJECT” for two allocentric spatial judgments, i.e. the “Allo-Allo block”; “BODY-OBJECT” for an egocentric and then an allocentric spatial judgment, i.e. the “Ego-Allo block”; “OBJECT-BODY” for an allocentric and then an egocentric spatial judgment, i.e. the “Allo-Ego block”). Each trial started with a jittered inter-stimulus interval (ISI) that randomly varied between 3 and 5 s per trial (Fig. 3b). The blocks were presented in counterbalanced order across participants and with a jittered inter-block interval (IBI) that randomly lasted between 25 and 35 s (Fig. 3c).

### Eye-tracking apparatus, data acquisition and pre-processing

Pupillary measurements methods, experimental design and data processing were performed according to the most recent literature on pupillometry guidelines ^46,47^.

Continuous measurements of both left and right pupil changes were recorded by means of the wearable head-unit eye tracking system Tobii Pro Glasses 2, with a sampling rate of 100 Hz. The eye-tracking system was equipped with 4 cameras (2 *per* eye) with a resolution of 240 × 960 pixels, and 12 infrared illuminators (6 *per* eye) built into the frame of the glasses above and below the eyes. The eye-tracking system was also equipped with a scene camera located above the nose pad of the glasses to record the scene with a full HD resolution of 1920 × 1080 pixels at 25 frames per second (fps). Before each recording, a calibration of the eye-tracking system was carried out for each participant through a calibration card provided by the manufacturer and the Tobii Glasses Controller Software. To calibrate the eye-tracking system, participants were required to fix at the calibration card held at arm’s length. No participant was excluded due to lack of or incorrect calibration.

The Tobii Pro Lab software was used to manage and extract pupil-size data. Pupil size analyses were carried out on the entire trial duration, encompassing both learning and testing phases, henceforth defined as “time of interest” (ToI). The pre-processing stream began with the blink reconstruction phase: missing and invalid data (due to blink or partially obscured pupil by eyelids) were recovered through linear interpolation. The subtractive baseline correction method ^29^ was used to remove trial-to-trial fluctuations in pupil size. Baseline pupil dilation was calculated as the mean value recorded during the first 50 ms after the triad appeared in the learning phase ^45^. This baseline was then subtracted from all subsequent measurements. Finally, the mean pupil dilation variation (in mm) for each experimental condition (Ego-Ego, Allo-Allo, Ego-Allo, Allo-Ego) was then estimated by block-averaging mean pupil-size data within conditions.

### Statistical analysis

*Pupil-size data.* A repeated-measure ANOVA with Spatial Judgments (Ego-Ego, Allo-Allo, Ego-Allo, Allo-Ego) as a four-level within-variable on the mean pupil dilation variation (in mm) was carried out. Moreover, to analyse the distinct contribution of the egocentric and allocentric judgments in the switching/non-switching conditions, two separate ANOVAs for Switching and Non-Switching task on the mean pupil dilation variation (in mm) of first and second judgments were carried out (Ordered Judgments) as for the behavioural data. To also measure the switch cost, a One-Way ANOVA with Conditions (Non-Switching, Switching) as a two-level within-variable was carried out on the mean pupil dilation variation (in mm) averaged between non-switching (Ego-Ego, Allo-Allo) and switching (Ego-Allo, Allo-Ego) conditions.

*Behavioural data.* Two repeated-measure ANOVAs with Spatial Judgments (Ego-Ego, Allo-Allo, Ego-Allo, Allo-Ego) as a four-level within-variable on the average of the first and second spatial judgments were carried out on mean accuracy (0–1) and RT (sec), respectively. Each participant’s mean accuracy was calculated as the percentage of correct responses (wrong = 0, correct = 1, range of scores for each condition = 0–18). This was obtained by dividing the total number of correct responses for combined conditions (e.g., Ego-Ego or Allo-Ego) by 18 (i.e., the maximum accuracy). Furthermore, to analyse the distinct contribution of the egocentric and allocentric judgments in the switching/non-switching conditions, two separate ANOVAs for each Switching and Non-Switching task on the distinct mean accuracy (score range = 0–9) and RT (sec) of first and second judgments were carried out (Ordered Judgments). For the Switching task, a One-Way ANOVA was carried out with Ordered Judgments (First-Ego, Second-Allo, First-Allo, Second-Ego) as a four-level within-variable. As regards the Non-Switching task, a One-Way ANOVA was carried out with Ordered Judgments (First-Ego, Second-Ego, First-Allo, Second-Allo) as a four-level within variable. Finally, to measure the switch cost, two separate ANOVAs with Conditions (Non-Switching, Switching) as a two-level within-variable were carried out on mean accuracy (score range = 0–36) and RT (in sec) averaged between non-switching (Ego-Ego, Allo-Allo) and switching (Ego-Allo, Allo-Ego) conditions. Lastly, to exclude speed–accuracy trade-off effects, a Pearson correlation between accuracy and response time for each condition (Switching: Ego-Allo, Allo-Ego; Non-Switching: Ego-Ego, Allo-Allo) was carried out.

Overall, the Tukey HSD test was used to analyse post hoc effects, and the magnitude of the significant effects were expressed by partial eta squared ($\:{\eta\:}_{p}^{2}$).

### Results

#### Eye-tracking data: pupil dilation variation

**Fig. 4:**
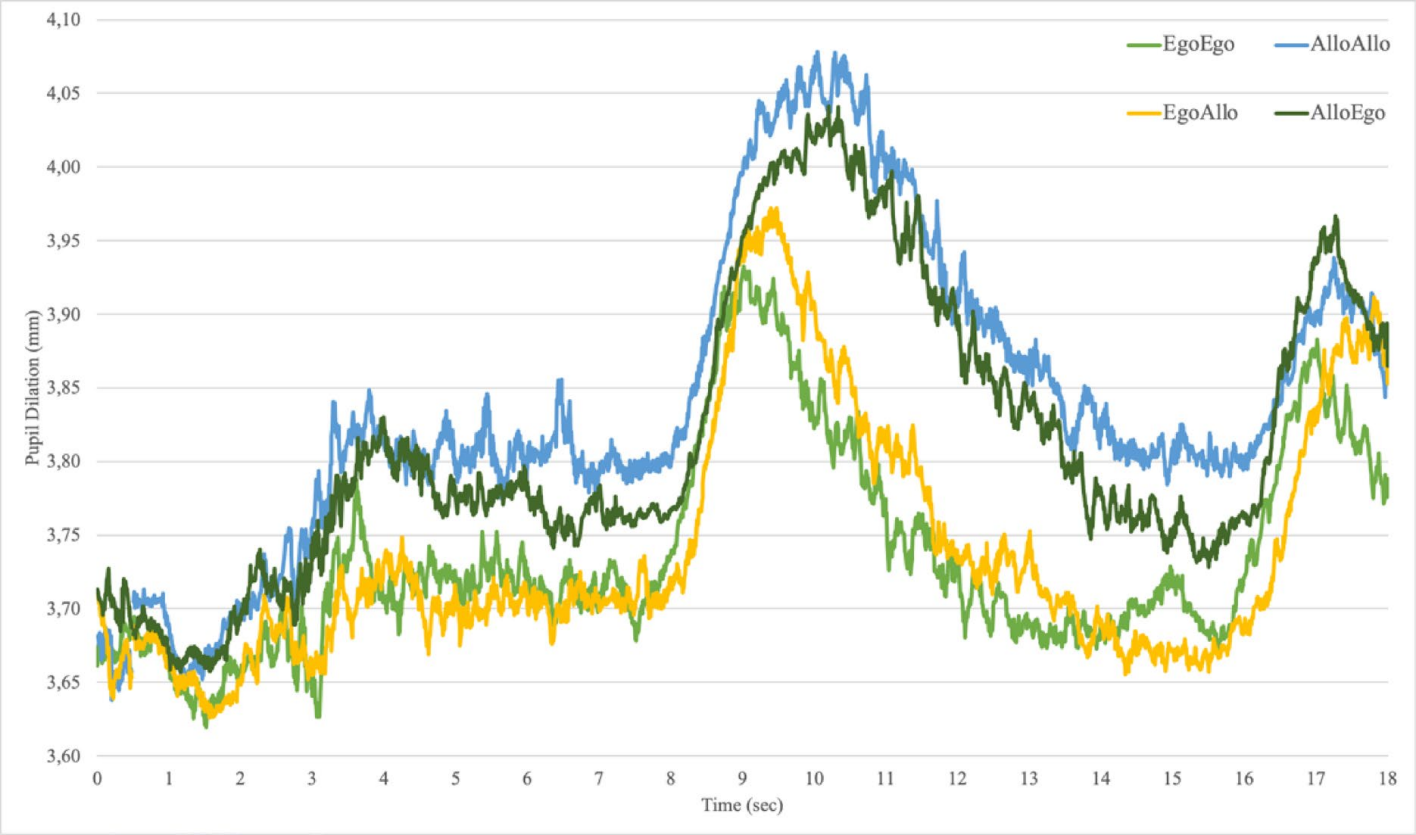
The plot depicts for descriptive purposes the time course of the pupil dilation (in mm) averaged across trials during learning (0–7 s) and testing (7–18 s) phases as a function of Switching (Ego-Allo in yellow, Allo-Ego in dark green) and Non-Switching (Ego-Ego in light green, Allo-Allo in blue) spatial conditions.

For descriptive purposes, in Fig. 4 the pupil dilation (in mm) averaged across trials as a function of non-switching (Ego-Ego, Allo-Allo) and switching (Ego-Allo, Allo-Ego) conditions is plotted.

**Fig. 5:**
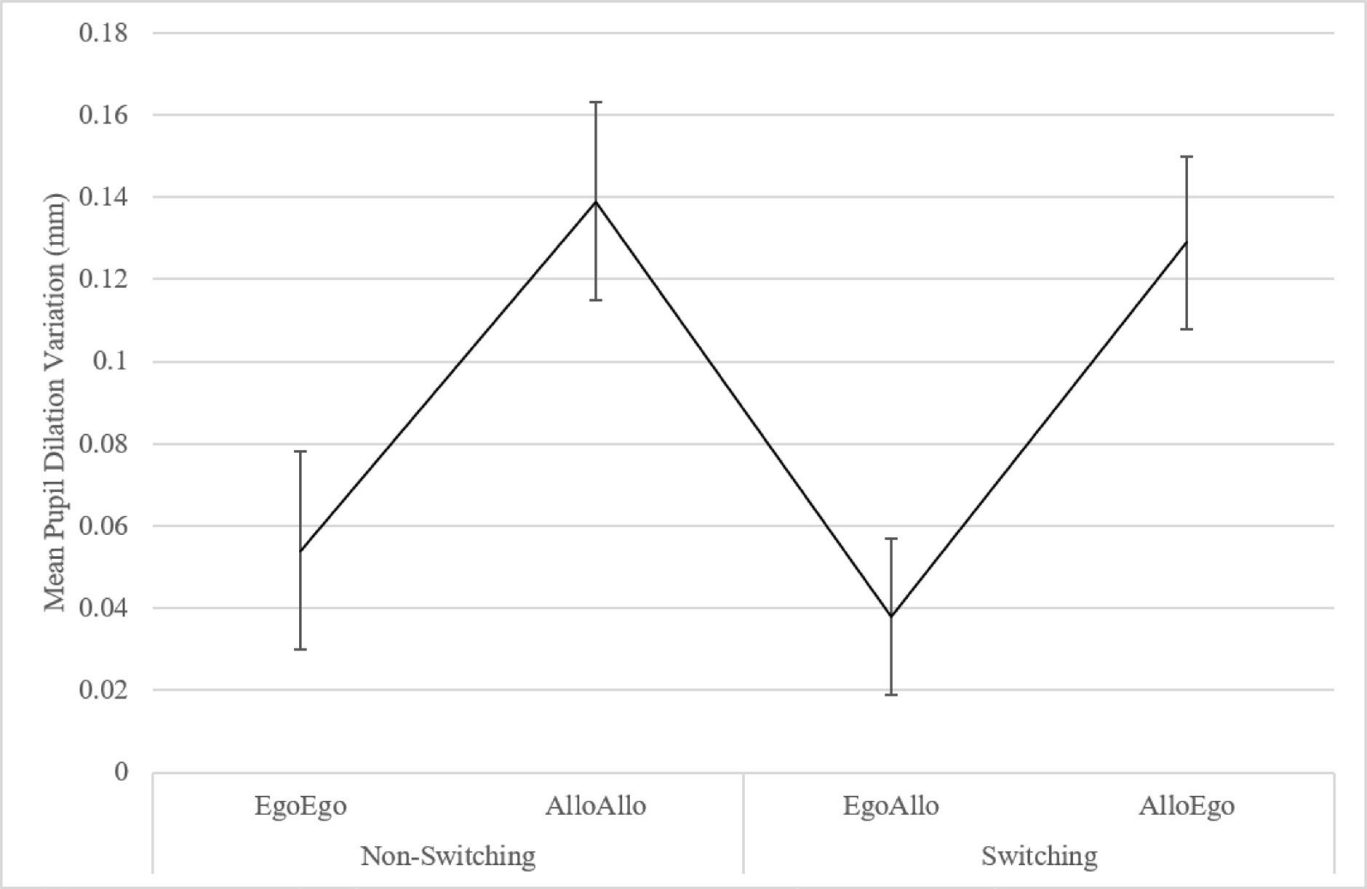
Mean Pupil Dilation Variation (mm) in Non-switching (Ego-Ego, Allo-Allo) and Switching (Ego-Allo, Allo-Ego) conditions. Error bars correspond to standard errors of the mean (S.E.M.).

The RM ANOVA on the mean pupil dilation variations revealed, a main effect of the Spatial Judgments (F(3, 57) = 13.7120, *p* < 0.0001, $\:{\eta\:}_{p}^{2}$ = 0.4192). The post-hoc analysis showed that when participants were required to provide allocentric-based spatial judgments in both switching (Allo-Ego, M = 0.1285, SE = 0.0212) and non-switching (Allo-Allo, M = 0.1393, SE = 0.0244) conditions, a greater pupil dilation variation was observed with respect to egocentric-based spatial judgments in both switching (Ego-Allo, M = 0.0379, SE = 0.0190) and non-switching (Ego-Ego, M = 0.0541, SE = 0.0241) conditions (at least *p* = 0.0263) (Fig. 5). No other significant differences emerged.

##### Distinct spatial judgments: switching task

**Fig. 6:**
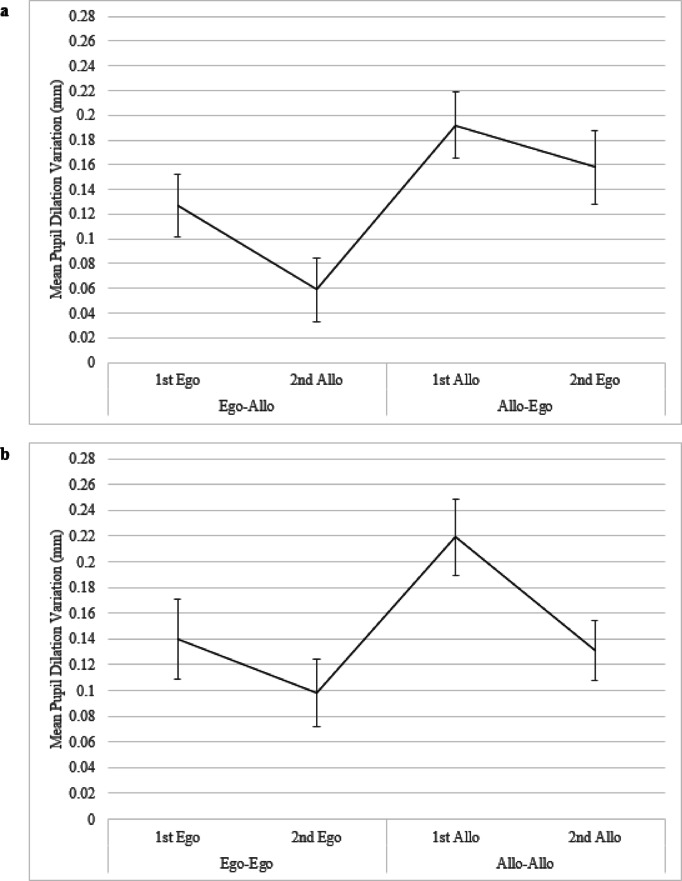
**a** Mean Pupil Dilation Variation (mm) in Ego-Allo (1st Ego, 2nd Allo) and Allo-Ego (1st Allo, 2nd Ego) Non-switching conditions. Error bars correspond to standard errors. **b** Mean Pupil Dilation Variation (mm) in Ego-Ego (1st Ego, 2nd Ego) and Allo-Allo (1st Allo, 2nd Allo) Non-switching conditions. Error bars correspond to standard errors of the mean (S.E.M.)

A main effect of the Ordered Judgments for pupil dilation variation emerged for the switching task (F(3, 57) = 14.2210, *p* < 0.0001, $\:{\eta\:}_{p}^{2}$ = 0.4281) (Fig. 6a). The post-hoc analysis showed that the mean pupil dilation variation was greater for the allocentric judgments of the Allo-Ego combination (1st Allo M = 0.1921, SE = 0.0269) compared to both spatial judgments of the Ego-Allo combination (1st Ego M = 0.1274, SE = 0.0255, 2nd Allo M = 0.0591, SE = 0.0259) (respectively *p* = 0.0087 and *p* = 0.0004). The post-hoc analysis revealed also that the allocentric judgment of the Ego-Allo combination (2nd Allo) resulted in a smaller pupil dilation variation compared to the egocentric judgments of the Allo-Ego (2nd Ego M = 0.1580, SE = 0.0299) one (*p* = 0.0009). Furthermore, the egocentric judgment of the Ego-Allo combination (1st Ego) elicited a greater pupil dilation variation compared to the allocentric judgment of the Ego-Allo combination (2nd Allo) (*p* = 0.0084).

##### Distinct spatial judgments: non-switching task

A main effect of the Ordered Judgments for pupil dilation variation emerged for the non-switching task (F(3, 57) = 15.2283, *p* < 0.0001, $\:{\eta\:}_{p}^{2}$ = 0.4449) (Fig. 6b). The post-hoc analysis showed that the mean pupil dilation variation was greater for the first allocentric judgments of the Allo-Allo combination (1st Allo M = 0.2188, SE = 0.0299) compared to all the other spatial judgments (2nd Allo M = 0.1313, SE = 0.0227; 1st Ego M = 0.1401, SE = 0.0309; 2nd Ego M = 0.0977, SE = 0.0257) (respectively *p* < 0.0001, *p* = 0.0026, *p* = 0.0006). No other significant difference emerged.

##### Non-switching vs. Switching conditions

The ANOVA did not reveal any significant difference between Conditions (F < 1) on mean pupil dilation variation.

#### Behavioural results: accuracy

Descriptive analyses of accuracy and response time for the switching/non-switching conditions are reported in Table 1.

**Table 1:** Mean values (M) and standard deviations (SD, in italics in the table) of the accuracy (0–1) and of the RT (sec) for the non-switching and switching conditions.

Visuospatial memory tasks							
Accuracy (Mean %)				Response time (sec)			
Non-switching		Switching		Non-switching		Switching	
Ego-Ego	Allo-Allo	Ego-Allo	Allo-Ego	Ego-Ego	Allo-Allo	Ego-Allo	Allo-Ego
M (*SD*)	M (*SD*)	M (*SD*)	M (*SD*)	M (*SD*)	M (*SD*)	M (*SD*)	M (*SD*)
0.83 (*0.11*)	0.56 (*0.12*)	0.71 (*0.14*)	0.56 (*0.11*)	1.55 (*0.25*)	1.95 (*0.21*)	1.86 (*0.26*)	1.93 (*0.27*)

#### Mean spatial judgments

A significant main effect of the Spatial judgments emerged (F(3, 57) = 34.2212, *p* < 0.0001, $\:{\eta\:}_{p}^{2}$ = 0.6430) with the Ego-Ego spatial judgements (M = 0.83, SE = 0.03) being more accurate than all other spatial judgements (at least *p* = 0.0005). Moreover, the Ego-Allo spatial judgments were more accurate than Allo-Allo and Allo-Ego ones (respectively *p* = 0.0002, *p* = 0.0049).

#### Distinct spatial judgments: switching task

A main effect of the Ordered Judgments for the switching task appeared (F(3, 57) = 33.3820, *p* < 0.0001, $\:{\eta\:}_{p}^{2}$ = 0.6373). The post-hoc analysis showed that the egocentric judgments of the Ego-Allo combination (1st Ego M = 0.89, SE = 0.03) were more accurate than the allocentric judgments of the same combination (2nd Allo M = 0.54, SE = 0.04) (*p* < 0.0001), and more accurate than both spatial judgments of the Allo-Ego combination (1st Allo M = 0.51, SE = 0.03; 2nd Ego M = 0.60, SE = 0.03) (both *p* < 0.0001). Moreover, in the Allo-Ego combination the egocentric judgments (2nd Ego) were more accurate than the allocentric ones (1st Allo) (*p* = 0.05).

#### Distinct spatial judgments: non-switching task

A main effect of the Ordered Judgments emerged for the non-switching task (F(3, 57) = 38.7604, *p* < 0.0001, $\:{\eta\:}_{p}^{2}$ = 0.6711). The post-hoc analysis showed that the egocentric judgments of the Ego-Ego combination (1st Ego M = 0.92, SE = 0.02; 2nd Ego M = 0.73, SE = 0.04) were more accurate than both allocentric judgments of the Allo-Allo combination (1st Allo M = 0.56, SE = 0.03; 2nd Allo M = 0.56, SE = 0.04) (at least *p* = 0.0108). Moreover, within the Ego-Ego condition, the first egocentric judgments (1st Ego) were more accurate than the second ones (2nd Ego) (*p* < 0.0001).

#### Non-switching vs. switching conditions

A significant difference between Conditions emerged (F(1, 19) = 12.0367, *p* = 0.0026, $\:{\eta\:}_{p}^{2}$ = 0.3878), due to Non-Switching judgments (M = 0.69, SE = 0.02) being more accurate than Switching ones (M = 0.63, SE = 0.02).

#### Behavioural results: response time

##### Mean spatial judgments

A main effect of Spatial Judgments was found (F(3, 57) = 30.0857, *p* < 0.0001, $\:{\eta\:}_{p}^{2}$ = 0.6129) with Ego-Ego spatial judgements (M = 1.5460, SE = 0.0554) being faster than all the other judgements (*p* < 0.001).

##### Distinct spatial judgments: switching task

A main effect of the Ordered Judgments emerged for the switching task (F(3, 57) = 19.1834, *p* < 0.0001, $\:{\eta\:}_{p}^{2}$ = 0.5024). The post-hoc analysis showed that egocentric judgments of the Ego-Allo (1st Ego M = 1.7154, SE = 0.0763) and Allo-Ego (2nd Ego M = 1.7912, SE = 0.0816) combinations were faster than allocentric spatial judgments of the Ego-Allo (2nd Allo, M = 2.1285, SE = 0.0559) and Allo-Ego (1st Allo M = 2.0967, SE = 0.0535) combinations (at least *p* = 0.0034).

##### Distinct spatial judgments: non-switching task

A main effect of the Ordered Judgments emerged for non-switching task (F(3, 57) = 7.3310, *p* < 0.0001, $\:{\eta\:}_{p}^{2}$ = 0.2784). The post-hoc analysis showed that egocentric judgments of the Ego-Ego combination (1st Ego M = 1.5310, SE = 0.0575, 2nd Ego M = 1.5660, SE = 0.0628) were faster than allocentric judgments of the Allo-Allo combination (1st Allo M = 1.9136, SE = 0.1187, 2nd Allo M = 1.8858, SE = 0.0608) (at least *p* = 0.0481).

##### Non-switching vs. switching conditions

The ANOVA revealed a significant difference between Conditions (F(1, 19) = 23.2146, *p* < 0.0001, $\:{\eta\:}_{p}^{2}$ = 0.5499), due to Switching judgments (M = 1.8963, SE = 0.0554), being slower than non-Switching ones (M = 1.7458, SE = 0.0425).

#### Correlation

The correlation analysis did not reveal any significant correlations between the mean accuracy and response times of non-switching (Ego-Ego, Allo-Allo) and switching (Ego-Allo, Allo-Ego) spatial judgments. No speed–accuracy trade-off effects were observed.

## Discussion

In all our daily activities, we need to specify spatial information according to egocentric and allocentric reference systems and, likewise, to switch between egocentric- and allocentric-based spatial representations. The present study aimed to investigate, along with behavioural measures, the task-evoked pupillary response supposed to reflect the cognitive load behind switching and non-switching processes between egocentric and allocentric frames of reference in order to clarify the entity of the switch cost in such visuo-spatial processes.

Overall, both task-evoked pupillary response and behavioural results showed neat differences in egocentric- and allocentric-based processes in both non-switching and switching conditions. Concerning the behavioural results, participants were faster and more accurate in providing non-switching egocentric-based spatial judgments (Ego-Ego) compared to all the other spatial judgments. Moreover, when the first anchor point was allocentric in both switching (Allo-Ego) and non-switching (Allo-Allo) conditions, participants were slower and less accurate compared to switching conditions when the first anchor point was egocentric (Ego-Allo). Overall, the behavioural results were in line with previous studies showing that spatial representations anchored to an egocentric reference frame are facilitated compared to those anchored to an allocentric reference frame ^6,7,18,22^. Notably, the behavioural results confirmed that translating between egocentric and allocentric spatial reference frames produces a switch cost as theorized by Rogers and Monsell ^27^ (see also ^26,29^). Indeed, the analysis of switching vs. non-switching conditions showed that participants were slower and less accurate in switching (Ego-Allo, Allo-Ego) rather than non-switching (Ego-Ego; Allo-Allo) spatial judgments.

Consistent with the behavioural results and in line with the expectations, the analysis of the task-evoked pupil responses showed that in non-switching conditions the allocentric spatial judgments (i.e., Allo-Allo) elicited a greater pupil dilation variation than egocentric (i.e., Ego-Ego) ones. These results suggest that providing spatial judgments according to an allocentric reference frame leads to a higher cognitive load compared to providing spatial judgments according to an egocentric reference frame. Furthermore, the increased cognitive load due to an allocentric spatial representation was equally evident in switching conditions. Indeed, in line with our hypothesis, the results showed that when participants were required to provide switching spatial judgments starting from an allocentric towards an egocentric reference frame (i.e., Allo-Ego), a greater pupil dilation variation was observed compared to switching judgments starting from an egocentric towards an allocentric reference frame (Ego-Allo). These results demonstrated that adopting and maintaining an allocentric rather than an egocentric spatial representation is more resource-demanding probably due to the fact that the representation in memory of inter-objects relationships is less automatic compared to the representations of body-objects ones that are pivotal for body-environment interactions (^48^; see also ^6,7,18,22,32,39,42^).

Notably, our results demonstrated that, when switching between spatial representations, starting from an allocentric instead of an egocentric reference frame has a different weight in terms of cognitive load. In this regard, the analysis of the distinct spatial judgments confirmed that an allocentric-based spatial representation prior to switch elicited a higher cognitive load than an egocentric-based spatial representation: participants exhibited a greater pupil dilation variation during the first allocentric judgments of the Allo-Ego combination (1st Allo) compared to both spatial judgments of the Ego-Allo combination (1st Ego, 2nd Allo). Conversely, the egocentric judgments of the Ego-Allo and Allo-Ego combination (i.e., 1st Ego, 2nd Ego) elicited a greater pupil dilation variation compared to the allocentric judgments of the Ego-Allo combination (i.e., 2nd Allo). Overall, these results suggest that switching from egocentric to allocentric representations (Ego-Allo) would result in a less resource demanding process compared to the opposite switching process (i.e., Allo-Ego). This pupillometric pattern consistently reflects what has been achieved by previous behavioural and neuroimaging research ^6,7,18–25^. Indeed, considering that the pupillary reflex is thought to be related to changes in arousal and mental activity modulated by the locus-coeruleus noradrenalin system (LC-NA) ^37^, our findings are consistent with the idea that visuospatial switching processes between reference frames rely also on the LC-NA system ^16,17^, besides to posteromedial structures ^15^ and fronto-parieto-temporal brain regions ^18^.

Noteworthy, within the Ego-Allo combination the task evoked pupil response of the second allocentric judgment (2nd Allo) was narrower compared to those of the first egocentric judgments (1st Ego). However, such difference of the second spatial judgments evoking a narrower pupil dilation variation compared to the first ones was not evident within the Allo-Ego combination. These results suggest that starting from an egocentric reference frame would facilitate the switch towards the subsequent allocentric one. Conversely, starting from an allocentric reference frame would, in turn, affect the switch towards the subsequent egocentric one ^6^. Although the pupillometric data indicated a continuous effortful processing, the behavioural data showed that the second egocentric spatial judgment of the Allo-Ego combination (2nd Ego) was faster and more accurate than the first allocentric one (1st Allo). It has been suggested that once an allocentric representation is formed, this would include also the subject’s position as one of the locations ^49^. This entails that participants should easily retrieve egocentric representations from allocentric ones. Arguably, despite participants performed more accurately and faster in the second egocentric judgment compared to the first allocentric one of the Allo-Ego combination, the sustained task-evoked pupil response reflected an underlying cognitive processing that remained effortful. Probably this could be attributed to the cost associated with adopting and starting from an allocentric reference frame. Consistently, our data showed a facilitation when spatial representations were egocentric-based versus allocentric-based and the egocentric judgments were facilitated when preceding rather than following an allocentric judgment. It is therefore possible that our paradigm, in which a specific type of encoding was required and the triad to be learned was presented for two seconds in front of the participant, facilitated egocentric representation because the body provided a stable anchoring ^4^.

These results expand those of previous studies with clinical populations in which it has been shown that allocentric-to-egocentric switching can impair egocentric performance ^6^, suggesting that allocentric encoding can interfere with subsequent egocentric retrieval. Our results enrich this evidence by showing that this form of interference may be covert in the behavioural performance in healthy population, but can be detected measuring the task-evoked pupil response(s). In other words, starting from an allocentric judgment seems to have exerted an influence on the subsequent egocentric response, that was not captured by accuracy or response times but emerged in the physiological index of cognitive effort measured by means of cognitive pupillometry.

Overall, these results reinforce the idea that egocentric and allocentric reference frames are not mutually exclusive ^13^. In this context, such interdependency resulted in a contamination of one frame of reference over the other (i.e., allocentric bias; ^50–52^). More precisely, the results showed that, when switching from an egocentric to allocentric reference frame, the process requires low cognitive resources. On the contrary, when switching from an allocentric reference frame (Allo-Ego), this resulted in a high resource demanding process that in turn produces a carry-over effect also on the subsequent body-centered representations. This so-called allocentric contamination could be explained by the fact that the allocentric penalizes the retrieval of the egocentric representation (Allo-Ego) in contrast to when the egocentric representation precedes the allocentric representation (Ego-Allo) ^6,7,18,22^.

Furthermore, it should be noted that, contrary to our expectations, the results did not reveal a difference in the task-evoked pupillary responses between Switching and Non-Switching conditions. Such lack of difference could be due to the fact that, regardless of the condition, providing an allocentric-based spatial judgment would provoke an increase in the cognitive load. Generally, these results support the idea that during visuo-spatial switching and non-switching processes the cognitive load varies depending on the anchor point/reference system from which one starts.

## Conclusions

This study measured for the first time the cognitive load behind visuospatial switching/non-switching processes between reference frames by means of cognitive pupillometry. Specifically, a greater pupil dilation was found in non-switching condition when only allocentric spatial judgments were required, and in switching condition between reference frames when the first anchorage point was allocentric (from Allo to Ego). Such results indicate that the adoption of an allocentric based spatial strategy is more resource demanding compared to the adoption of an egocentric-based one, in both switching and non-switching visuospatial processes. These findings align with previous work showing that the adoption of an allocentric reference frame is more cognitively demanding than an egocentric one ^36,42^. The key finding, however, is that switching from an allocentric to an egocentric reference frame (Allo-Ego) elicited a significant greater pupil dilation than the opposite direction (Ego-Allo). This asymmetry cannot be explained solely by the intrinsic cost of allocentric processing. If that were the case, we would expect comparable pupil dilation in any condition requiring allocentric spatial judgments, regardless of whether they occurred in the first or second part of the trial. Therefore, although allocentric representations are cognitively demanding, our findings show that these demands are significantly influenced by the switching dynamics.

## Data Availability

The data that support the findings of this study are available from the corresponding author upon reasonable request.
